# The Effects of E-Cigarette Use on Alcohol and Marijuana Abuse Symptoms in an Ethnically Diverse Sample of Young Adults

**DOI:** 10.3390/ijerph182413159

**Published:** 2021-12-14

**Authors:** Pallav Pokhrel, Taha Elwir, Hannah Mettias, Crissy T. Kawamoto, Nabin Oli, Scott K. Okamoto

**Affiliations:** 1 Population Sciences in the Pacific Program, University of Hawai‘i Cancer Center, University of Hawaii at Manoa, 701 Ilalo St., Honolulu, HI 96813, USA; elwir9@hawaii.edu (T.E.); ckawamoto@cc.hawaii.edu (C.T.K.); sokamoto@hpu.edu (S.K.O.); 2 John A. Burns School of Medicine, University of Hawai‘i at Manoa, 651 Ilalo St., Honolulu, HI 96813, USA; hmettias@hawaii.edu; 3 Kokua Kalihi Valley Comprehensive Family Services, 2239 N School St., Honolulu, HI 96819, USA; drnabinoli@yahoo.com; 4 School of Social Work, College of Health & Society, Hawai‘i Pacific University, 1 Aloha Tower Drive, Honolulu, HI 96813, USA

**Keywords:** e-cigarette, cigarette, alcohol, marijuana, Asian, Pacific Islander, substance abuse

## Abstract

Background: We examined e-cigarette use as a prospective predictor of alcohol and marijuana abuse symptoms in a sample consisting of Native Hawaiian and Other Pacific Islander (NHPI), Filipino, Asian (i.e., Japanese, Chinese, Korean), and White young adults. NHPI represent a highly vulnerable group with regard to substance use and are severely understudied. Methods: Data were collected from 1463 young adults (M age = 22.2, SD = 3.2; 59.5% women) enrolled across community colleges in Hawai‘i at two time-points six months apart. Results: Higher frequency of e-cigarette use at baseline was predictive of higher alcohol (B = 0.06, SE = 0.02, *p* < 0.01) and marijuana (B = 0.06, SE = 0.02, *p* < 0.01) use problems at six-month follow up, adjusting for baseline cigarette smoking, problem alcohol/marijuana use, sensation seeking, and demographic variables. Ethnicity was found to significantly moderate the relationship between baseline e-cigarette use and problem marijuana use later, such that White and NHPI ethnicities were particularly vulnerable to the effects of e-cigarette use on problem marijuana use. Conclusion: NHPI are often combined with Asians in national surveys, which obfuscates the higher risks faced by NHPI compared with groups that are routinely classified as Asians (e.g., Chinese, Japanese, Filipinos). The current research highlights the NHPI’s vulnerability in terms of the effects of e-cigarette use on marijuana and alcohol abuse symptoms.

## 1. Introduction

At present, e-cigarette use is the most common medium of nicotine consumption among young people in the United States [1,2]. Among adults, e-cigarette use is most common among young adults (e.g., 18–25-year-olds) [3]. Approximately 50% of current e-cigarette users among young adults appear to never have smoked a cigarette [3]. Although e-cigarettes are considered less harmful to health than cigarettes, growing evidence links e-cigarette use with a range of respiratory disorders, after adjusting for the effects of cigarette smoking [4]. Furthermore, there are concerns about the effects of e-cigarette use on initiation and escalation of other substance use [5,6,7].

Cigarette smoking is considered a risk factor for alcohol and other substance use disorders [8,9,10]. Nicotine in cigarettes is likely to potentiate the physiological effects of alcohol, marijuana, and other substances [11,12,13]. E-cigarettes are marketed and used as alternatives to cigarettes [14,15]. E-cigarette use is positively correlated with cigarette smoking and has been associated with increased levels of alcohol, marijuana, and other substance use [5,7,16,17,18]. In addition, although used mainly for nicotine consumption, e-cigarettes are also used to vape marijuana [19,20,21]. Currently, little is known about how e-cigarette use may be associated with substance use disorders after accounting for the effects of cigarette smoking. Given e-cigarettes’ dominant presence among young people, this is a significant gap in the literature. As with cigarette smoking, the nicotine consumed through e-cigarettes may potentiate the effects of alcohol and marijuana. Moreover, in the case of marijuana use, vaping marijuana may directly contribute to problem marijuana use, as consumption of marijuana through vaping may be more discreet such that excessive use is not conspicuous to others.

Another area in which currently there is limited information is how e-cigarette use may differentially affect problem substance use among vulnerable ethnic groups. Native Hawaiian and other Pacific Islanders (NHPI), who are indigenous to the Hawaiian and other Pacific islands, have historically been at increased risks for tobacco and other substance use [22]. For most of modern history, NHPI have lived under circumstances of cultural and political colonization, economic deprivation, and related historical trauma and ethnic discrimination [23]. Socio-economic problems, historical trauma, and ethnic discrimination have contributed significantly to the vulnerability experienced by NHPI in terms of high substance use related morbidity and mortality [22,24,25]. Unfortunately, most of the disparities faced by NHPI in this regard and others have been overlooked because of the tendency in national surveys to group lower risk Asians (e.g., Japanese, Chinese, Korean) and Pacific Islanders together. Oftentimes, studies conducted on the mainland U.S. lack access to a sizeable sample of NHPI, because of which, such studies are unable to examine NHPI as a separate group. Studies conducted in Hawai‘i have the advantage of focusing on NHPI as a separate group and comparing them with other groups, including White and Asian.

The population of Hawai‘i is ethnically diverse, with 25% White, 23% part or full NHPI, 16% Filipino, 26% other Asian (e.g., Japanese, Chinese, Korean), and 10% others [26]. Filipinos are another group, who, commonly classified as Asians in national studies, are understudied in public health research [27]. According to the Hawai‘i-based health and socio-economic indicators, Filipinos appear to be intermediate in vulnerability compared with NHPI and other Asians; such that, Filipinos may be less vulnerable than NHPI and more vulnerable than other Asians [28]. This applies to tobacco and other substance use as well [28]. The first notable migration of Filipinos into the Hawaiian Islands occurred in the early 20th century when people from the Philippines were recruited to work in the sugar plantations of Hawai‘i [29]. For generations, the Filipinos in Hawai‘i mainly functioned as migrant workers at lower strata of the socio-economic hierarchy. As a result, historically, the Filipinos in Hawai‘i have faced socio-economic deprivation and ethnic discrimination [29], which may have contributed to the current health disparities faced by the group [27].

The present study was designed to examine the prospective effects of e-cigarette use on problem marijuana and alcohol use across NHPI, Filipino, other Asian (e.g., Japanese, Chinese, Korean; Asian, henceforth), and White young adults recruited from two-year or community colleges in Hawai‘i. Thus, in addition to answering questions about the effects of e-cigarette use on problem marijuana and alcohol use, the study sought to examine whether such effects are stronger for more vulnerable groups such as the NHPI.

## 2. Materials and Methods

### 2.1. Procedures

Data were collected from seven community (two year) colleges across the state of Hawai‘i. All seven colleges belonged to the same university system. Email addresses of all 18–29-year-olds enrolled across the seven colleges were obtained from the registrar’s office. A random sample of e-mail addresses was selected from the list and invited via email to complete a screener survey. The screener survey included questions on respondents’ age, gender, current college status (e.g., enrollment status), and cigarette smoking and e-cigarette use status. The main inclusion criteria were age and community college status. The focus on community colleges was motivated by the fact that community colleges tend to attract different types of hard-to-reach populations who may be at high risk for tobacco use such as racial/ethnic minorities and individuals pursuing blue-collar professions [30]. Information on gender and smoking and e-cigarette use status was used to monitor the sample composition. Based on our past experience, women and never cigarette smokers are prompt to respond to an invitation to take an online survey. Therefore, after a certain point in data collection, invitations were managed such that women and never cigarette smokers or e-cigarette users were not invited to complete the screener survey. The objective was to obtain a sample that was reflective of the population of young adult community college students in Hawai‘i.

The response rate to the e-mail invitations was 58%. That is, of those who received email invitation to complete the screener survey (approximately 6000), 58% completed the screener survey. This response rate is similar to response rates we have obtained in past studies based on the same method of recruitment [21]. Potential respondents received up to five e-mail reminders to complete the screener survey. They completed a brief online consent form before proceeding to complete the screener survey, which was programmed on Qualtrics.

Women who had never smoked a cigarette were over-represented among those who were recruited through email invitation. In order to recruit more men and cigarette smokers/e-cigarette users, we attempted to supplement the email method with classroom-based recruitment. Close to 45 classrooms across academic disciplines at a college, including vocational and technical training programs, were randomly selected at each college. Instructors of these classes were approached for permission to visit their classrooms for recruitment purposes. However, during the course of the in-class recruitment, pandemic mitigation efforts began to be enforced and in-person instruction was gradually switched to an online mode. Because of this, we were able to access only the colleges on O‘ahu, where 75% of Hawai‘i’s population resides. Across the four colleges on O‘ahu, on average, 43 classes were accessed. Students accessed at the classroom completed the screener survey in the classroom. On average, 85% of the students approached in the classrooms across the four colleges completed the screener survey.

The screener survey collected through e-mail invitation and in the classroom were processed exactly the same way. Research staff contacted the eligible students by phone. The study was explained in detail to the potential participants, including the informed consent procedure. Those still interested in participating in the study after the phone meeting were sent a link to the consent form. The form was programmed on Qualtrics. Respondents were asked to read the form and express consent by clicking a button labeled “I agree to participate in this study.” Once the respondents provided consent, they were taken to a form, also programmed on Qualtrics, that asked them to provide their detailed contact information, including contact information for their close family and friends. Once consent and contact form completion was verified, research staff emailed participants a link to the baseline survey, programmed also on Qualtrics.

**Figure 1:**
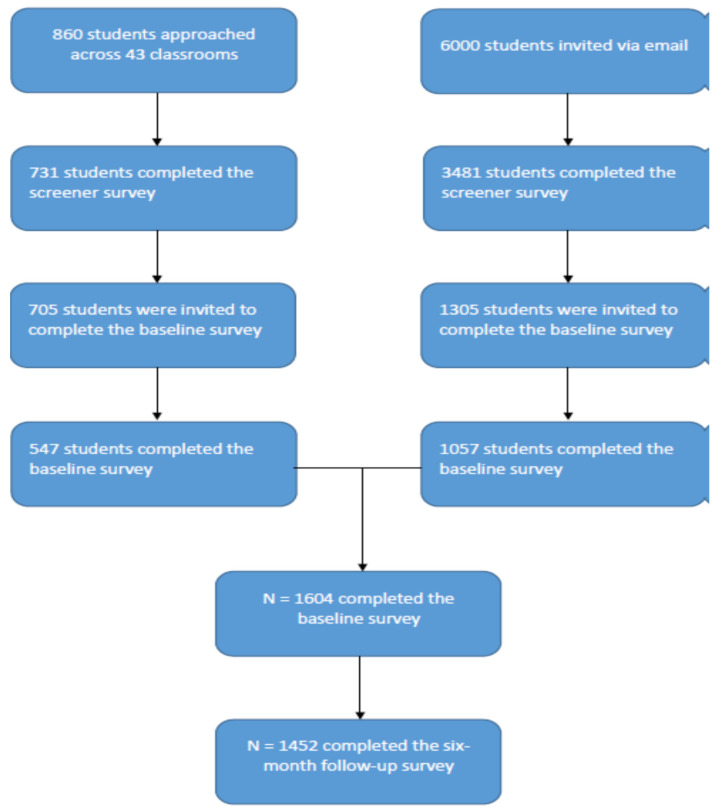
Flow-chart representing the recruitment procedures. Note that the analyses in the present study excluded “Other” ethnicity, because of which the analysis sample sizes are different than those presented in the figure.

Figure 1 depicts the recruitment procedures in the form of a flow-chart. A total of 1604 participants completed the baseline survey. Of these, 1057 had been recruited through e-mail invitation and 547 through classroom visits. All 1604 of the baseline participants were contacted again six months later for a follow-up survey. Of the 1604 baseline participants, 1452 completed the follow-up survey (retention rate = 90%). When the 10% of the participants who were lost to follow-up were compared with the complete baseline sample on baseline measures of the variables considered in the current study, no statistically significant differences were detected. For the purpose of ethnic comparison, current analyses excluded cases that were not classified as White, Asian, Filipino, or NHPI. As a result, the baseline sample size relevant for the current analyses was 1463, and the follow-up sample size was 1329.

### 2.2. Measures

#### 2.2.1. Demographics

Single items were used to obtain information on age, sex, household income, and number of hours worked per week for pay. To determine ethnicity, participants were asked “What is your ethnic background?” and were provided with a list of ethnicities common in Hawai‘i and the U.S. The question was asked in two different ways. The first question asked participants to refer to the list and “check all that apply.” The second question asked participants to choose the ethnic background that they identified with most. The response to the second question was utilized to assign mixed-ethnicity individuals to a particular racial/ethnic category.

#### 2.2.2. Sensation Seeking

Sensation seeking was assessed with the Brief Sensation Seeking Scale (8-items; α = 0.91) [31]. Example items include, “I would like to explore strange places,” “I prefer friends who are excitingly unpredictable,” and “I like wild parties”. Each item was scored on a 5-point scale ranging from “Never,” to “Usually.”

#### 2.2.3. Current Cigarette, E-Cigarette, and Marijuana Use

Current e-cigarette use, cigarette smoking, and marijuana use were assessed in terms of past-30-day use. For example, “During the last 30 days (1 month), on how many days did you use an electronic cigarette (e-cigarette) or a similar vaping device?” (8-point scale: “0 days,” “1–2 days,” “3–5 days,”…, “All days”). Past-30-day marijuana use was assessed in terms of general marijuana use and marijuana vaping, with separate items.

#### 2.2.4. Alcohol Abuse Symptoms

Problem alcohol use was assessed using the commonly used Alcohol Use Disorders Identification Test (AUDIT; 10 items; α = 0.82) [32]. Each of the 10 items was scored on a 5-point scale ranging from 0 to 4. Example items include, “During the past year, how often have you failed to do what was normally expected of you because of drinking?”; “How often do you have six or more drinks on one occasion?” A problem alcohol use scale was created by summing up the responses across the 10 items.

#### 2.2.5. Marijuana Abuse Symptoms

Problem marijuana use was measured with the Cannabis Abuse Screening Test (CAST; 6 items) [33,34,35]. Each item was scored on a 5-point scale ranging from 0 to 4. Example items include, “Have you ever smoked cannabis before midday?”; “Have you ever smoked cannabis when you were alone?” A problem marijuana use scale was created by summing up the responses across the 6 items. CAST has been well validated among young people representing both inpatient and general populations [33,34,35].

### 2.3. Data Analysis

Data were analyzed on SAS version 9.3 (SAS Institute Inc., Cary, NC, U.S.A.). Ethnic differences in continuous and categorical variables were tested using general linear models and chi-square tests, respectively. The associations between baseline current e-cigarette use and problem alcohol and marijuana use at six-month follow-up were tested using multiple linear regression models. The model testing the association between e-cigarette use at baseline and problem alcohol use at six-month follow-up adjusted for demographic variables (i.e., age, sex, ethnicity, household income, hours worked per week for pay) and baseline problem alcohol use and current cigarette smoking. The model testing the association between e-cigarette use at baseline and problem marijuana use at six-month follow-up adjusted for demographic variables, baseline problem marijuana use, baseline marijuana vaping, and baseline cigarette smoking.

Next “ethnicity X e-cigarette use” interaction effects were examined to test the ethnic differences in the effects of baseline e-cigarette use on later problem alcohol and marijuana use. Steps outlined by Aiken and West [36] were followed. First, all continuous various in the previous regression models were standardized (mean = 0, standard deviation = 1). Next, three “ethnicity X baseline e-cigarette use” interaction terms were created by dummy coding three ethnic groups (Asian, Filipino, NHPI) with reference to White. To test interaction effects, the interaction terms were entered into the regression models tested above.

## 3. Results

### 3.1. Participants

Table 1 summarizes the baseline characteristics of the participants (N = 1463) in terms of their demographic and substance use characteristics. Twenty-six percent of the sample were Asian, 29% Filipino, 25% White, and 20% NHPI. Statistically significant ethnic differences were found across variables. White participants tended to be older than participants in other groups. NHPI young adults were more likely to work over 40 h per week compared with other groups. White participants tended to report highest levels of substance use and problem marijuana and alcohol use, followed by NHPI. Differences were marked for past-30-day marijuana use in particular, with 39% White participants reporting past 30-day-use, followed by 23% NHPI, 15% Filipino, and 7% Asian. Thirty percent (*n* = 443) participants reported current e-cigarette use at baseline. Of these, 78% reported usually using nicotine e-liquid, 7% reported they usually used non-nicotine e-liquid, and 15% did not report whether or not they vaped nicotine.

### 3.2. Associations of Baseline E-Cigarette Use with Future Problem Alcohol and Marijuana Use

Table 2 shows the results of the regression analysis examining the effects of baseline e-cigarette use on problem alcohol use at six-month follow-up. Higher e-cigarette use at baseline was a statistically significant predictor of higher problem alcohol use six months later, controlling for ethnicity and other demographic variables, sensation seeking, baseline problem alcohol use, and baseline cigarette smoking. Ethnicity was not an independent predictor of problem alcohol use.

Table 3 shows the results of the regression analysis examining the effects of baseline e-cigarette use on problem marijuana use at six-month follow-up. Higher e-cigarette use at baseline was a statistically significant predictor of higher problem marijuana use six months later, controlling for ethnicity and other demographic variables, sensation seeking, baseline problem marijuana use, baseline marijuana vaping frequency, and baseline cigarette smoking. Relative to White ethnicity, NHPI ethnicity had a statistically significant inverse main effect on problem marijuana use.

### 3.3. Moderation by Ethnicity

Ethnicity was not found to moderate the effects of e-cigarette use on problem alcohol use. With problem alcohol use as the dependent variable, “Ethnicity X baseline e-cigarette use” interaction terms were not found to be statistically significant for “NHPI X e-cigarette use” [unstandardized regression coefficient (B) = 0.02, standard error (SE) = 0.06, *p* = 0.70], “Filipino X e-cigarette use” (B = −0.10, SE = 0.06, *p* = 0.06), and “Asian X e-cigarette use” (B = −0.08, SE = 0.05, *p* = 0.16).

**Figure 2:**
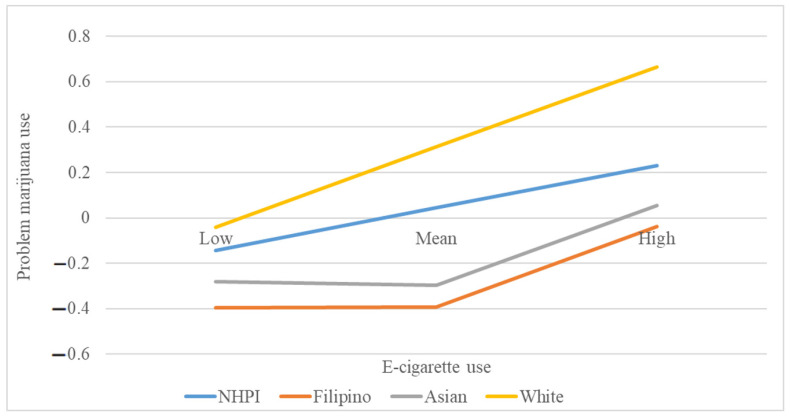
Relationship between baseline e-cigarette use and future problem marijuana use as moderated by ethnicity. (NHPI: Native Hawaiian and other Pacific Islander. Low, Mean, and High denote levels of e-cigarette use at mean, one standard deviation unit lower than the mean (low), and one standard deviation higher than the mean (high)).

With problem marijuana as the dependent variable, however, there were statistically significant “ethnicity X baseline e-cigarette use” interaction effects for “NHPI X e-cigarette use” (B = −0.13, SE = 0.05, *p* = 0.01), “Filipino X e-cigarette use” (B = −0.14, SE = 0.05, *p* < 0.01), and “Asian X e-cigarette use” (B = −0.11, SE = 0.05, *p* = 0.03). Figure 2 graphically illustrates the statistically significant interaction effect of ethnicity on the association between baseline e-cigarette use and problem marijuana use at six-month follow-up. As can be seen, the rate of change in problem marijuana use as a function of e-cigarette use varied across the four ethnic groups. The rate of change was steepest for Whites, followed by NHPI. Asian, Filipino, and NHPI compared the same amount of change in current e-cigarette use at baseline for the three ethnic groups, resulting in the highest amount of change in problem marijuana use for NHPI. The differences in rates of change were greater at higher levels of e-cigarette use.

## 4. Discussion

This study attempted to answer whether current e-cigarette use is prospectively associated with problem marijuana and alcohol use among young adults and whether such association is moderated by ethnicity. More specifically, we attempted to understand whether certain vulnerable ethnic groups are more at risk for experiencing the adverse effects of e-cigarette use on problem marijuana and alcohol use. The present data indicated that e-cigarette use is prospectively associated with both problem alcohol and marijuana use, even after adjusting for potential confounders such as cigarette smoking and baseline problem alcohol and marijuana use, respectively. Ethnicity was found to moderate the effects of e-cigarette use on problem marijuana use but not problem alcohol use, such that White young adults were most vulnerable to the effects of e-cigarette use on problem marijuana use, followed by NHPI young adults. Filipino young adults were least at risk.

To our knowledge, this is the first study to have examined the prospective effects of e-cigarette use on problem alcohol or marijuana use among young adult college students. Thus, the present findings regarding the effects of higher e-cigarette use on higher problem use of alcohol and marijuana may be some of the first evidence suggesting that e-cigarette use exacerbates alcohol and marijuana abuse symptoms. The fact that the majority of our current e-cigarette users at baseline reported being regular nicotine users may suggest that nicotine may explain the effects of e-cigarette use on increased alcohol or marijuana use among young adults. Although the present analysis is insufficient to make this deduction with any degree of certainty, the current findings emphasize the need to understand the mechanisms of the effects of e-cigarette use on problem alcohol and marijuana use. Understanding such mechanisms is especially important, given the recent rise in marijuana use among young adults [37]. Note that the current data showed an effect of e-cigarette use on increased marijuana use problems, even after adjusting for marijuana vaping.

The finding that White young adults were found to be most vulnerable to the effects of e-cigarette use on problem marijuana use may be explained by the ethnic differences in age-related factors that were unaccounted for in the current analysis. The White subsample in the present sample was significantly older than other groups. Older young adults are likely to have had longer histories of alcohol drinking and marijuana use. In addition, older young adults may have easier access to alcohol and marijuana because of experience as well as social networks, formed over a longer period of time, that are conducive to substance use. Likewise, age-related factors may explain Filipino young adults’ lower vulnerability to the effects of e-cigarette use. The Filipino subsample showed the youngest average age in the current sample. However, the present study does not have the necessary data to test these propositions. Future studies may need to pay closer attention to the age-related processes.

As expected, NHPI young adults showed higher vulnerability to the effects of e-cigarette use on problem marijuana use than Asian or Filipino young adults. This evidence highlights how NHPI young adults are at greater risk for experiencing the negative consequences of e-cigarette when compared with other groups that are routinely combined into a single “Asian/Pacific Islander” category. More research is needed to understand why NHPI young adults experience differential adverse impact of e-cigarette use. Research shows that NHPI are likely to experience higher levels of stressors and mental health symptoms [22,24,25]. Higher stress or negative affect is known to make individuals more sensitive to the positive reinforcement due to substance use [38]. It is possible that e-cigarette use enhances the pleasant effects of marijuana use more strongly for NHPI compared with other Asian or Filipino group. However, this is speculative and needs to be tested in future research. Regardless, it appears that NHPI may greatly benefit from e-cigarette use prevention and cessation interventions.

There are limitations to this study. First, although attempts were made to access a random, representative sample of community college students in Hawai‘i, due to COVID, in person recruitment had to be aborted and islands other than O‘ahu could not be accessed for classroom-based recruitment. This may have introduced some selection bias into the current sample. Second, because the current sample was recruited from community colleges, the sample may differ from a random community sample of young adults. For example, reflecting the general gender imbalance in college-going population, the current sample included more women than men. Therefore, there is some concern that the findings may not generalize to non-college young adults or four-year college students. However, it should be noted that community college demography includes higher proportions of several vulnerable groups that four-year college demography does not, such as ethnic/racial minorities and individuals being prepared for careers in blue-collar professions [30]. Lastly, the current data did not include sufficient information to be able to understand why certain ethnic groups may be more vulnerable to the effects of e-cigarette use on problem marijuana use.

## 5. Conclusions

Despite the limitations, the current study is significant for highlighting the effects of e-cigarette use on increased future alcohol and marijuana abuse symptoms. More importantly, the current study showed that certain ethnic groups (e.g., NHPI) might be more vulnerable than others to the adverse impact of e-cigarette use on problem marijuana use. More research is needed to understand why that is so. Such information would help control e-cigarette misuse. Lastly, the present findings emphasize the e-cigarette use prevention and treatment needs of White community college students and NHPI students.

## 6. Implications

The current findings have three important implications. First, the effects of e-cigarette use on increased problem alcohol and marijuana use amplify the urgent need for e-cigarette use prevention and cessation programs targeted at young adults, especially college students. Second, the stronger effects of e-cigarette use on problem alcohol and marijuana use among White students in the current sample imply that White young adults who attend community colleges may be most at risk for the harmful consequences of e-cigarette use. Future research is needed to better understand this phenomenon. Lastly, NHPI college students appear to be at increased for the adverse effects of e-cigarette use, in terms of e-cigarette’s role in exacerbating the symptoms of alcohol and marijuana use, compared with groups commonly classified as Asians. Thus, there may be a need for a culturally grounded e-cigarette use prevention and treatment programs designed specifically for NHPI.

## Figures and Tables

**Table 1:** Baseline participant characteristics by ethnicity.

		All (N = 1463)	NHPI (*n* = 290)	Filipino (*n* = 423)	Asian (*n* = 382)	White (*n* = 368)	
			Mean (SD)/%				Range
Age ***		22.2 (3.2)	22.3 (3.2)	21.4 (3.0)	22.2 (3.1)	23.0 (3.3)	18–30
Sex							
	Men	40.5%	39.7%	42.5%	43.2%	36.1%	
	Women	59.5%	60.3%	57.5%	56.8%	63.9%	
Household income ***							
	USD 0–19 K	22.8%	31.2%	18.7%	20.7%	22.7%	
	USD 20–39 K	23.3%	24.8%	20.0%	20.7%	27.8%	
	USD 40–69 K	23.6%	23.1%	24.1%	23.8%	23.3%	
	USD 70–99 K	11.9%	9.8%	14.6%	13.7%	9.2%	
	USD 100–119 K	10.5%	6.4%	13.7%	12.0%	8.8%	
	USD 120 K or Over	7.9%	4.7%	8.9%	9.0%	8.2%	
Hours/week worked for pay ***							
	0 h	24.1%	26.9%	24.2%	24.2%	21.9%	
	1–9 h	8.9%	6.6%	8.3%	9.7%	10.7%	
	10–19 h	20.6%	17.9%	23.3%	24.7%	15.3%	
	20–29 h	21.2%	19.7%	21.6%	20.7%	22.4%	
	30–39 h	10.7%	13.8%	10.0%	7.6%	12.3%	
	40 h	9.9%	6.6%	9.0%	9.5%	13.9%	
	Over 40 h	4.6%	8.6%	3.6%	3.7%	3.6%	
Current substance use							
	E-cigarette **	30.3%	34.8%	29.5%	23.8%	39.3%	
	Cigarette	10.0%	10.7%	9.2%	9.7%	14.4%	
	Marijuana ***	23.5%	23.4%	15.1%	7.0%	39.1%	
	Binge drinking ***	28.6%	31.4%	24.6%	23.3%	36.4%	
Problem use							
	Alcohol ***	4.2 (4.8)	4.8 (5.4)	3.6 (4.3)	3.5 (4.1)	5.1 (5.3)	0–25
	Marijuana ***	3.2 (4.9)	4.0 (5.2)	1.9 (3.7)	2.2 (3.9)	5.1 (5.9)	0–24

Note. SD = Standard deviation; NHPI = Native Hawaiian and Other Pacific Islander. (** *p* < 0.01, *** *p* < 0.001).

**Table 2:** The effects of baseline e-cigarette use on problem alcohol use at six-month follow-up.

Independent Variables	B (SE)
Age	0.05 (0.05)
Sex (men: 0, women: 1)	−0.02 (0.02)
Hours/week worked for pay	−0.01 (0.04)
NHPI	0.03 (0.02)
Filipino	−0.09 (0.06)
Asian	−0.04 (0.06)
T1 Sensation seeking	0.04 (0.02)
T1 current cigarette smoking	0.01 (0.02)
T1 problem alcohol use	0.65 (0.02) ***
T1 current e-cigarette use	0.06 (0.02) **

Note. B = Unstandardized regression coefficient, SE = Standard error, NHPI = Native Hawaiian and Other Pacific Islanders, T1 = baseline. (** *p* < 0.01, *** *p* < 0.001).

**Table 3:** The effects of baseline e-cigarette use on problem marijuana use at six-month follow-up.

Independent Variables	B (SE)
Age	0.05 (0.02) *
Sex (men: 0, women: 1)	0.12 (0.04) **
Hours/week worked for pay	−0.001 (0.02)
NHPI	−0.12 (0.06) *
Filipino	−0.09 (0.05)
Asian	−0.03 (0.05)
T1 Sensation seeking	0.06 (0.02) **
T1 current cigarette smoking	−0.04 (0.02)
T1 current marijuana vaping	0.01 (0.02)
T1 problem marijuana use	0.66 (0.02) ***
T1 current e-cigarette use	0.06 (0.02) **

Note. B = Unstandardized regression coefficient, SE = Standard error, NHPI = Native Hawaiian and Other Pacific Islanders, T1 = baseline. (* *p* < 0.05, ** *p* < 0.01, *** *p* < 0.001).

## Data Availability

The data relevant to the current analysis may be available from Pallav Pokhrel (ppokhrel@cc.hawaii.edu).
